# Parasites of domestic and wild animals in South Africa. L. Ixodid ticks infesting horses and donkeys

**DOI:** 10.4102/ojvr.v84i1.1302

**Published:** 2017-02-28

**Authors:** Ivan G. Horak, Heloise Heyne, Ali Halajian, Shalaine Booysen, Willem J. Smit

**Affiliations:** 1 Department of Veterinary Tropical Diseases, University of Pretoria, South Africa; 2 Parasites, Vectors and Vector-borne Diseases, Agricultural Research Council-Onderstepoort Veterinary Institute, South Africa; 3 Department of Biodiversity, University of Limpopo, South Africa; 4 Onderstepoort Veterinary Academic Hospital, University of Pretoria, South Africa

## Abstract

The aim of the study was to determine the species spectrum of ixodid ticks that infest horses and donkeys in South Africa and to identify those species that act as vectors of disease to domestic livestock. Ticks were collected opportunistically from 391 horses countrywide by their owners or grooms, or by veterinary students and staff at the Faculty of Veterinary Science, University of Pretoria. Ticks were also collected from 76 donkeys in Limpopo Province, 2 in Gauteng Province and 1 in North West province. All the ticks were identified by means of a stereoscopic microscope. Horses were infested with 17 tick species, 72.1% with *Rhipicephalus evertsi evertsi*, 19.4% with *Amblyomma hebraeum* and 15.6% with *Rhipicephalus decoloratus. Rhipicephalus evertsi evertsi* was recovered from horses in all nine provinces of South Africa and *R. decoloratus* in eight provinces. Donkeys were infested with eight tick species, and 81.6% were infested with *R. evertsi evertsi*, 23.7% with *A. hebraeum* and 10.5% with *R. decoloratus*. Several tick species collected from the horses and donkeys are the vectors of economically important diseases of livestock. *Rhipicephalus evertsi evertsi* is the vector of *Theileria equi*, the causative organism of equine piroplasmosis. It also transmits *Anaplasma marginale*, the causative organism of anaplasmosis in cattle. *Amblyomma hebraeum* is the vector of *Ehrlichia ruminantium*, the causative organism of heartwater in cattle, sheep and goats, whereas *R. decoloratus* transmits *Babesia bigemina*, the causative organism of babesiosis in cattle.

## Introduction

Despite the long association between horses, donkeys and humans in South Africa, it is strange that so little attention has been paid to the ixodid ticks with which they are infested. With the exception of surveys on the ticks that infest donkeys in Botswana (Mushi et al. 2003) and donkeys and horses in Ethiopia (Ferede et al. 2010; Kumsa et al. 2012), there are no comprehensive studies on the ticks that infest these animals in sub-Saharan Africa. The data that are available are fragmented in that they generally have to be garnered from publications or surveys in which horses and donkeys as well as other animals were examined for ticks.

In their extensive study of the zoogeography of the ixodid ticks of Tanzania, Yeoman and Walker (1967) identified ticks in collections made from multiple host species including four horses and six donkeys. *Amblyommma variegatum, Rhipicephalus appendiculatus, Rhipicephalus decoloratus, Rhipicephalus evertsi evertsi* and a tick identified as *Rhipicephalus sanguineus* were recovered from the horses and donkeys. In a similar study in Kenya, collections were made from 19 horses and 3 donkeys (Walker 1974). In addition to the aforementioned ticks, *Amblyomma gemma, Dermacentor rhinocerinus, Hyalomma rufipes, Rhipicephalus jeanelli, Rhipicephalus pulchellus, Rhipicephalus simus* (probably *Rhipicephalus preatextatus*) and a tick belonging to the *Haemapysalis leachi* group were identified. Norval and his co-workers in Zimbabwe examined collections from 39 horses and 11 donkeys and identified *Amblyomma hebraeum, H. rufipes, Haemapysalis truncatum, R. appendiculatus, R. decoloratus, R. evertsi evertsi, Rhipicephalus kochi, Rhipicephalus* sp. (near *Rhipicephalus punctatus*), *R. simus, Rhipicephalus turanicus* and *Rhipicephalus zambeziensis* in the collections from horses, and *A. hebraeum, H. rufipes, R. appendiculatus, R. decoloratus, R. evertsi evertsi* and *R. simus* in those from donkeys (Norval 1981, 1982, 1983; Norval & Mason 1981; Norval, Walker & Colborne 1982). In their book on the *Rhipicephalus* species of the world, Walker, Keirans and Horak (2000) record a total of 25 *Rhipicephalus* species and two subspecies on horses and 15 species and two subspecies on donkeys. Only two of these species, *R. appendiculatus* and *R. simus* and the two subspecies, *R. evertsi evertsi* and *R. evertsi mimeticus*, are present in South Africa, whereas the remainder occurs extralimitally.

Mushi et al. (2003) collected *A. hebraeum, Hyalomma* sp. and *R. evertsi evertsi* from 12 donkeys examined at monthly intervals over a period of 7 months in their study devoted to the parasites of donkeys in the Kgatleng District, Botswana. Ferede et al. (2010) collected *A. variegatum, H. rufipes, R. evertsi evertsi, R.* (*Boophilus*) spp., *Rhipicephalus muhsamae* and a tick they referred to as *R. sanguineus* from a total of 450 donkeys in a survey conducted in two districts in central Oromia Regional State, Ethiopia. In the same region of Oromia, Kumsa et al. (2012) collected *A. gemma, A. variegatum, H. rufipes, H. truncatum, R. decoloratus, R. evertsi evertsi* and *R. pulchellus* from 1168 horses distributed in highland, midland and lowland localities.

No serious effort was made to collect all the ticks present on the horses or donkeys in any of the abovementioned studies. In two southern African studies on the ticks of zebras, in which a few horses were included, Horak, Biggs and Reinecke (1984b) collected *H. rufipes, H. truncatum* and a large number of larvae, nymphs and adults of *Rhipicephalus evertsi mimeticus* from three horses in the Khomas Hochland of Namibia, whereas Horak, Knight and De Vos (1986) collected the larvae of *Amblyomma marmoreum* and of *Margaropus winthemi*, the adults of *Hyalomma glabrum, H. truncatum, Rhipicephalus follis* and *Rhipicephalus gertrudae* and all stages of development of *R. evertsi evertsi* and of *Rhipicephalus glabroscutatus* from two horses in the Mountain Zebra National Park in the Eastern Cape Province. It is interesting to note that tick collections have been made from more plains zebras (*Equus quagga*), Cape mountain zebras (*Equus zebra zebra*) and Hartmann’s mountain zebras (*Equus zebra hartmannae*) than from domesticated horses or donkeys in South Africa or Namibia (Horak et al. 1984b, 1986; Horak, De Vos & De Klerk 1984a).

The objectives of this study were to determine the species composition of ixodid ticks that infest horses and donkeys in South Africa and to place in context the diseases they can potentially transmit to these animals and other domestic livestock.

## Methods

Ticks were collected from horses in all nine provinces of South Africa by their owners or grooms or by veterinary nurses and students from animals resident in the paddocks at the Faculty of Veterinary Science, University of Pretoria. In addition, S.B. (University of Pretoria) collected ticks from horses presented for treatment at the university’s Equine Clinic. Ticks previously collected from two horses in the Mountain Zebra National Park (Horak et al. 1986) have been included for completeness sake. Ticks were collected by their owners or by A.H. and W.J.S. (University of Limpopo) from 73 donkeys in Limpopo Province, 2 in Gauteng Province and 1 in North West province with the consent of the owners. Although no attempt was made to make total collections, particular attention was paid to the head, shoulders, inner thighs, perianal region, tail brush and around the hooves. The ticks from each animal were placed in separate vials containing 70% ethyl alcohol or methylated spirits with a pencil-written label providing collection data. The ticks were identified and counted by the project leader I.G.H. (University of Pretoria) and H.H. (ARC-Onderstepoort Veterinary Institute) using a stereoscopic microscope. The distribution by province of the various tick species was illustrated using a mosaic format.

## Ethical considerations

Protocol V077/14 for the collection of ticks from horses and donkeys was submitted by I.G.H. and approved by the Research Committee and the Animal Ethics Committee of the Faculty of Veterinary Science, University of Pretoria.

## Results and discussion

### General

**FIGURE 1:**
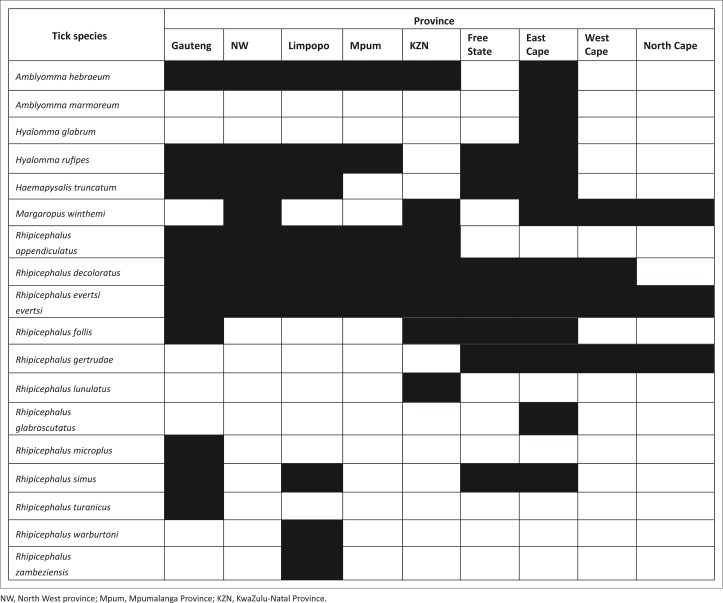
The provincial distribution of ixodid ticks that infest horses and donkeys in South Africa.

The species and numbers of ticks collected from 391 horses and 76 donkeys are summarised in Tables 1 and 2. The provincial distribution of the ticks collected from horses and donkeys is depicted in Figure 1. A total of 5327 adult and immature ticks were collected from horses and a total of 487 from donkeys. The horses were infested with 17 tick species and donkeys with 8 tick species. A single donkey was the only animal from which *Rhipicephalus warburtoni* was collected. The number of species and the total number of ticks collected from horses in the present survey were considerably higher than the 7 species and 917 ticks collected from horses in central Oromia, Ethiopia or those from donkeys in the Kgatleng District, Botswana, but similar to those of donkeys in central Oromia.

**TABLE 1:** Ticks collected from 391 horses throughout South Africa.

Tick species	Number infested	% infested	Number of ticks recovered			

			Larvae	Nymphs	Males	Females
*Amblyomma hebraeum*	76	19.4	0	41	245	146
*Amblyomma marmoreum*	2	0.5	14	0	0	0
*Hyalomma glabrum*	2	0.5	0	0	14	4
*Hyalomma rufipes*	41	10.5	0	0	77	47
*Hyalomma truncatum*	18	4.6	0	0	134	73
*Margaropus winthemi*	11	2.8	114 immatures	114 immatures	244	200
*Rhipicephalus appendiculatus*	41	10.5	0	79	213	171
*Rhipicephalus decoloratus*	61	15.6	0	0	70	371
*Rhipicephalus evertsi evertsi*	282	72.1	248 immatures	248 immatures	1464	782
*Rhipicephalus follis*	13	3.3	0	0	74	47
*Rhipicephalus gertrudae*	16	4.1	0	0	67	57
*Rhipicephalus glabroscutatus*	3	0.8	19 immatures	19 immatures	125	62
*Rhipicephalus lunulatus*	1	0.3	0	0	0	1
*Rhipicephalus microplus*	2	0.5	0	0	2	8
*Rhipicephalus simus*	20	5.1	0	0	47	34
*Rhipicephalus turanicus*	3	0.8	0	0	15	11
*Rhipicephalus zambeziensis*	1	0.3	0	0	5	2

**TABLE 2:** Ixodid ticks collected from 76 donkeys, mostly in Limpopo Province.

Tick species	Number infested	% infested	Number of ticks recovered			

			Larvae	Nymphs	Males	Females
*Amblyomma hebraeum*	18	23.7	0	85	15	3
*Hyalomma rufipes*	3	3.9	0	0	3	0
*Hyalomma truncatum*	10	13.2	0	0	9	2
*Rhipicephalus appendiculatus*	10	13.2	0	47	2	3
*Rhipicephalus decoloratus*	8	10.5	0	3	2	7
*Rhipicephalus evertsi evertsi*	62	81.6	43 immatures	43 immatures	220	36
*Rhipicephalus simus*	3	3.9	0	0	5	1
*Rhipicephalus warburtoni*	1	1.3	0	0	1	0

With the exception of *R. decoloratus* and *Rhipicephalus microplus*, of which the males are very small and engorged females large and *Rhipicephalus lunulatus*, of which only one female tick was collected, more male than female ticks of all species were collected. Twelve tick species were collected from horses in the Eastern Cape Province, 10 from horses and donkeys in Gauteng Province and 9 from horses and donkeys in Limpopo Province (Figure 1). *Rhipicephalus evertsi evertsi* was present on horses in every province, and *R. decoloratus* on these animals in every province except the Northern Cape (Figure 1).

### *Amblyomma* spp.

#### Amblyomma hebraeum

The bont tick, *A. hebraeum*, was the second most prevalent tick recovered from both horses and donkeys. This species was present on horses in each of the four northern provinces, as well as in KwaZulu-Natal and the Eastern Cape (Table 1; Figure 1). Its presence in these provinces is in agreement with its overall geographic distribution as mapped by Spickett (2013). The adults of *A. hebraeum* prefer large herbivores as hosts, whereas its immature stages and particularly the larvae infest the same hosts as the adults as well as smaller mammals, birds and tortoises (Horak et al. 1987; Horak, Golezardy & Uys 2007). The mean burden of adult ticks on 33 plains zebras examined in the Kruger National Park was 3 (Horak et al. 1984a), compared to 5 on the 76 horses infested with adult ticks in the present study. Horses on the same property as other livestock may thus serve as a reservoir of infestation and escape the sometimes rigorous acaricidal control regimens applied to cattle. *Amblyomma hebraeum* is the vector of *Ehrlichia ruminantium,* the causative organism of heartwater in cattle, sheep and goats and certain wildlife species (Neitz 1937; Norval & Horak 2004).

#### Amblyomma marmoreum

The South African tortoise tick, *Amblyomma marmoreum,* is widespread in South Africa, and whereas its adults and nymphs prefer leopard tortoises, *Stigmochyles pardalis,* as hosts, its larvae infest a wide range of mammals as well as the larger ground-frequenting birds and reptiles (Horak et al. 2006). The presence of larvae on the two horses and on four Cape mountain zebras examined in the Mountain Zebra National Park, where there are a large number of leopard tortoises, is thus not surprising (Horak et al. 2006).

### *Hyalomma* spp.

#### Hyalomma glabrum, Hyalomma rufipes *and* Hyalomma truncatum

The three *Hyalomma* species that are present in South Africa are drought and heat-tolerant and are generally present in those regions where these climatic conditions prevail. Previously, only two horses examined in the Mountain Zebra National Park were infested with *H. glabrum* (Horak et al. 1986). At that time, this tick was referred to as *Hyalomma marginatum turanicum*, but it was subsequently reinstated as *H. glabrum* (Apanaskevich & Horak 2006). In the present study, more horses were infested with *H. rufipes* than with *H. truncatum* but the intensity of infestation with the latter tick was higher (Table 1). In an earlier study carried out in Zimbabwe, 12 out of 39 horses were found to be infested with *H. rufipes* and the same number with *H. truncatum.* There too the intensity of infestation with the latter species was higher (Norval 1982). Donkeys in the present study were also infested with both species (Table 2). *Hyalomma rufipes* was collected from horses in six provinces and *H. truncatum* in five (Figure 1).

*Hyalomma rufipes* is the vector of *Babesia occultans*, the causative organism of benign babesiosis in cattle, and *H. truncatum* is the vector of *Babesia caballi*, the causative organism of equine piroplasmosis (De Waal 1990; Gray & De Vos 1981). Both species are two-host ticks and their immature stages feed on hares (Horak & Fourie 1991). Consequently, infection with *Babesia* spp. must pass transovarially from one generation of female ticks to the next generation of adult ticks.

#### Margaropus winthemi

The one-host winter horse tick, *M. winthemi,* has a scattered distribution associated with the cooler, higher altitude regions of South Africa (Howell, Walker & Nevill 1978). During July 1984, three zebras in the Mountain Zebra National Park were each found to be infested with more than 25 000 ticks in all stages of development (Horak et al. 1986). Penzhorn (1984) noted that of the 22 zebras in the park for which fairly accurate mortality dates were reported, 20 had died during the winter and that 19 of these deaths occurred between July and September. He regarded the late winter as a critical period for survival, probably because of the deteriorating condition of the forage and the cold weather. Horak et al. (1986) reported that the large burdens of *M. winthemi* during winter probably exacerbated the effects of the already harsh conditions. Although no *M. winthemi* were collected from horses in the Free State, large numbers have been collected from gemsbok in the Willem Pretorius Nature Reserve in the centre of the province (Fourie et al. 1991).

### *Rhipicephalus* spp.

#### Rhipicephalus appendiculatus

The provincial distribution of *R. appendiculatus* is similar to that of *A. hebraeum* (Spickett 2013; Figure 1). Forty-one of the 391 horses examined were infested, and the mean intensity of infestation on these animals was 5 males and 4 female ticks. In Zimbabwe 13 of 39 horses were infested and the mean intensity of infestation was 18 males and 13 female ticks (Norval et al. 1982). Ten of the 76 donkeys examined were infested. *Rhipicephalus appendiculatus* is a three-host tick of which the adults prefer large domestic and wild ruminants as hosts (Horak et al. 2007). It is the vector of *Theileria parva* as well as buffalo-derived *T. parva*, the causative organisms of East Coast fever and Corridor disease, respectively, in cattle (Norval & Horak 2004).

#### Rhipicephalus decoloratus *and* Rhipicephalus microplus

Excluding the western Free State, the Karoo and the Northern Cape Province, *R. decoloratus* is widespread throughout the rest of South Africa, whereas *R. microplus* is in the process of invading this region at the expense of *R. decoloratus* (Horak et al. 2009; Nyangiwe, Harrison & Horak 2013; Spickett 2013; Tønnesen et al. 2004). *Rhipicephalus decoloratus* was present on horses in every province excepting the Northern Cape in the present study (Figure 1). It prefers large ruminants as well as equids as hosts, whereas *R. microplus* is a cattle tick, but is in the process of adapting to other host species (Horak et al. 2009). *Rhipicephalus decoloratus* and *R. microplus* are one-host ticks and both transmit *Babesia bigemina*, whereas *R. microplus* also transmits *Babesia bovis,* the causative organisms of bovine babesiosis (De Vos, De Waal & Jackson 2004).

#### Rhipicephalus evertsi evertsi

With the exception of a large portion of the Northern Cape Province, *R. evertsi evertsi* is present throughout South Africa (Spickett 2013). This species was collected from horses in all nine provinces in the present study (Figure 1). Its overall distribution includes much of East Africa, eastern Sudan and several sub-Saharan countries in West Africa (Walker et al. 2000). More collections of *R. evertsi evertsi* have been made from horses, donkeys and plains zebras in the Afrotropical region than any other *Rhipicephalus* species (Walker et al. 2000). It is the dominant species on horses in South Africa and in Oramia Regional State in Ethiopia, and on donkeys in South Africa and Botswana (Kumsa et al. 2012; Mushi et al. 2003; Tables 1 and 2). The prevalence of *R. evertsi evertsi* on horses and donkeys in South Africa and Botswana was considerably higher than that on these animals in Ethiopia.

*Rhipicephalus evertsi evertsi* is a two-host tick whose adults attach in the perianal region and on the inner thighs and inguinal regions of equids and the immature stages in the external ear canals of these animals. Although the prevalence of infestation may be high, burdens of adult ticks are seldom large. Infestations with immature ticks may be very large. For instance, a mean of more than 850 larvae and nymphs have been collected from the external ear canals of 33 plains zebras in the Kruger National Park (Horak et al. 1984a).

*Rhipicephalus evertsi evertsi* transmits *B. caballi* and *Theileria equi,* the causative organisms of equine piroplasmosis (De Waal & Potgieter 1987; Norval & Horak 2004), and *Theileria separata,* the causative organism of ovine theileriosis (Jansen & Neitz 1956). It also transmits *Anaplasma marginale*, the causative organism of anaplasmosis in cattle (Potgieter 1981). Engorging *R. evertsi evertsi* females secrete a paralysis-inducing toxin that affects lambs born during spring in the highveld regions of Mpumalanga, the eastern Free State and the north-eastern region of the Eastern Cape Province (Gothe 1981).

#### Rhipicephalus follis, Rhipicephalus gertrudae *and* Rhipicephalus simus

With the exception of North West and Mpumalanga provinces, one or more of these three species within the ‘*R. simus*’ group of ticks were present on horses in every province. *Rhipicephalus follis* was found in the mountainous or higher altitude regions mainly in the eastern half of the country, *R. gertrudae* in the semi-arid or winter-rainfall regions with dry summers with *R. simus* in the warmer, moister lower altitude regions of the country (Walker et al. 2000).

#### Rhipicephalus glabroscutatus

The distribution of *R. glabroscutatus* stretches from the Albany Thicket biome in the Eastern Cape Province through the Fynbos biome to the West Coast National Park. It is a two-host tick, with all stages of development feeding around the hooves and below the fetlocks of its hosts. Secondary bacterial infection of its attachment sites leads to foot abscesses and lameness, particularly in Angora goats farmed in the Albany Thicket biome (MacIvor & Horak 1987).

### Species of which few were collected

*Rhipicephalus lunulatus* is a fairly rare species in South Africa and is present in the warmer moist regions in the east of the country. Its distribution is widespread in Zimbabwe, East and West Africa (Walker et al. 2000). There is considerable controversy concerning the identity of the tick known as *R. turanicus*. The tick referred to by this name in South Africa is not necessarily morphologically similar to ticks with the same name elsewhere, and further studies are required to establish its true identity. The distribution of *R. warburtoni* is more widespread than that plotted for it in the central and south-western Free State by Walker et al. (2000). Its presence has been confirmed in the north of Limpopo Province (Harrison, Bown & Horak 2011) as *R.* sp. (near *warburtoni*) and the collection of a male tick from a donkey is further proof of its presence there. *R. zambeziensis* is present in the Limpopo Valley and adjoining areas (Norval et al. 1982), and its morphology, hosts and seasonal abundance are similar to those of *R. appendiculatus*. It is also a vector of East Coast fever (Lawrence, Norval & Uilenberg 1983).

## Conclusion

Horses examined countrywide were found to be infested with a large variety of tick species of which *R. evertsi evertsi* was the most widespread. The prevalence of this tick is potentially important because it is the vector of the causative organisms of equine piroplasmosis. Various other tick species collected from the horses are important vectors of diseases in domestic cattle. Donkeys in Limpopo Province were infested with eight tick species of which *R. evertsi evertsi* was the most prevalent.
